# Molecular Analysis and Reclassification of NSD1 Gene Variants in a Cohort of Patients with Clinical Suspicion of Sotos Syndrome

**DOI:** 10.3390/genes14020295

**Published:** 2023-01-22

**Authors:** Barbara Testa, Giuseppina Conteduca, Marina Grasso, Massimiliano Cecconi, Francesca Lantieri, Chiara Baldo, Alessia Arado, Laura Andraghetti, Michela Malacarne, Donatella Milani, Domenico Coviello, Sotos Collaborative Group

**Affiliations:** 1 Laboratory of Human Genetics, IRCCS Istituto Giannina Gaslini, 16147 Genoa, Italy; 2 Dipartimento di Scienze della Salute, Sezione di Biostatistica, Università degli Studi di Genova, 16132 Genoa, Italy; 3 Unità di Pediatria ad Alta Intensità di Cura, Fondazione IRCCS Cà Granda, 20122 Milan, Italy

**Keywords:** Sotos syndrome, *NSD1*, variants of uncertain significance, NGS

## Abstract

Sotos syndrome is a rare genetic disorder caused by haploinsufficiency of the *NSD1* (nuclear receptor binding SET domain containing protein 1) gene. No clinical diagnostic consensus criteria are published yet, and molecular analysis reduces the clinical diagnostic uncertainty. We screened 1530 unrelated patients enrolled from 2003 to 2021 at Galliera Hospital and Gaslini Institute in Genoa. *NSD1* variants were identified in 292 patients including nine partial gene deletions, 13 microdeletions of the entire *NSD1* gene, and 115 novel intragenic variants never previously described. Thirty-two variants of uncertain significance (VUS) out of 115 identified were re-classified. Twenty-five missense *NSD1* VUS (25/32, 78.1%) changed class to likely pathogenic or likely benign, showing a highly significant shift in class (*p* < 0.01). Apart from *NSD1*, we identified variants in additional genes (*NFIX, PTEN, EZH2, TCF20, BRWD3, PPP2R5D*) in nine patients analyzed by the NGS custom panel. We describe the evolution of diagnostic techniques in our laboratory to ascertain molecular diagnosis, the identification of 115 new variants, and the re-classification of 25 VUS in *NSD1*. We underline the utility of sharing variant classification and the need to improve communication between the laboratory staff and the referring physician.

## 1. Introduction

Sotos syndrome (SoS) (OMIM 117550) is a rare genetic disorder whose prevalence is estimated to be 1:14,000 live births [1,2]. It is characterized by pre- and postnatal overgrowth (height and/or head circumference ≥98th percentile), advanced age compared to chronological age, delayed psychomotor development of varying degrees, and typical craniofacial anomalies such as macrocephaly, prominent forehead, downslanting palpebral fissures, and a pointed chin [1,2,3].

The growth pattern is especially increased in the early years of childhood [1]. Phenotypic overlap with other overgrowth syndromes exists, in particular with Weaver and Malan syndromes [4,5].

From the literature data, facial dimorphism is the most stringent criterion for clinical diagnosis [6,7,8,9].

However, no clinical diagnostic consensus criteria have yet been published for SoS and clinical evaluation and expertise can be different from center to center.

Clinical diagnosis can be difficult because of the wide variability of the features and the presence of cases described in the literature as “Sotos like” [10,11,12].

Molecular analysis of the *NSD1* gene (nuclear receptor binding SET domain containing protein 1) reduces diagnostic uncertainty in patients with suspected SoS and allows for diagnosis to be confirmed.

The *NSD1* gene (MIM 606681) is mapped to 5q35.2–q35.3 and consists of 23 exons, the first of which is noncoding. The open reading frame starts in the second exon, is 8088 bp long, and encodes 2696 amino acids. There are different known transcripts of *NSD1* [13,14,15]. Recently, we described two novel short *NSD1* isoforms that are expressed in both healthy individuals and in SoS patients [16].

NSD1 contains multiple functional domains including SET domain (Su(var) 3–9, Enhancer of Zeste and Trithorax) (SET) and SET-associated (SAC) domains that together mediate the histone methyltransferase activity of NSD1; a C5HCH and five plant homeodomains (PHDs), which are implicated in chromatin regulation and are zinc finger–like motifs characterized by cysteine and histidine residues as well as two proline-tryptophan-tryptophan-proline (PWWP) domains that may mediate protein–protein interactions and that are often found in proteins that act at the chromatin level [17,18].

NSD1 also contains two nuclear receptor interaction domains, NID-L and NID+L, which are typical of those found in nuclear receptor corepressors and coactivators, respectively [19].

The presence of these distinctive domains suggests that NSD1 is a histone-lysine N-methyltransferase that acts as a transcriptional intermediary factor capable of both negatively and positively influencing transcription, depending on the cellular context [13].

The NSD1 protein is also involved in the transcriptional silencing of developmentally regulated genes during embryogenesis [18].

Recently, gene set enrichment analysis showed that *NSD1* mutations induce the altered expression of long noncoding RNAs and genes controlling the G2/M checkpoint involved in neoplastic differentiation [20].

SoS is caused by a wide spectrum of pathogenic variants (truncating, missense, splice-site variants, partial gene deletions, and 5q35 microdeletions) that result in haploinsufficiency of the *NSD1* gene [5,21].

More than 500 variants in the *NSD1* gene are reported in the Human Gene Mutation Database (HGMD) and in the Leiden Open Variation Database (LOVD).

The large majority of *NSD1* abnormalities occur de novo and there are very few familial cases with autosomal dominant inheritance [22].

It has further been shown that there are some ethnic differences in the prevalence of different types of mutations. In particular, microdeletions involving the *NSD1* gene are very frequent in Japanese patients with SoS [23,24], and this finding is not frequently observed in non-Japanese populations, where intragenic point mutations are highly prevalent [7,24,25,26,27,28,29].

In this study, we report on the molecular analysis of the *NSD1* gene performed for diagnostic purposes on 1530 unrelated patients enrolled from 2003 to 2021 at Galliera Hospital (2003–2017) in the Human Genetics Laboratory, recently moved to Gaslini Institute (2018–2021) in Genoa.

We explicate the evolution of over 18 years of diagnostic activity in SoS in our diagnostic laboratory, describe 115 *NSD1* new variants never previously reported in the literature, detail the re-classification of 25 missense VUS, and identified nine patients carrying a pathogenetic variants in different genes from *NSD1*.

## 2. Materials and Methods

### 2.1. Patients

A total of 1530 Caucasian subjects with molecular investigation performed between June 2003 and June 2021 were recruited. This sample includes patients with clinical suspicion of Sotos syndrome, Sotos-like cases, and some cases of non-specific overgrowth that did not meet all the classical Sotos clinical inclusion criteria, since the activity was performed in the diagnostic laboratory part of the National Health System (NHS).

Informed consent and clinical data were obtained from all patients.

Clinical inclusion criteria for *NSD1* gene testing included the following conditions:

1. Typical facial “gestalt”;
2. Height above the 97th percentile (overgrowth);
3. Head circumference above the 97th percentile (macrocephaly);
4. Learning disability;
5. Congenital anomalies or malformations.

### 2.2. History of the Diagnostic Analysis Workflow

**Figure 1:**
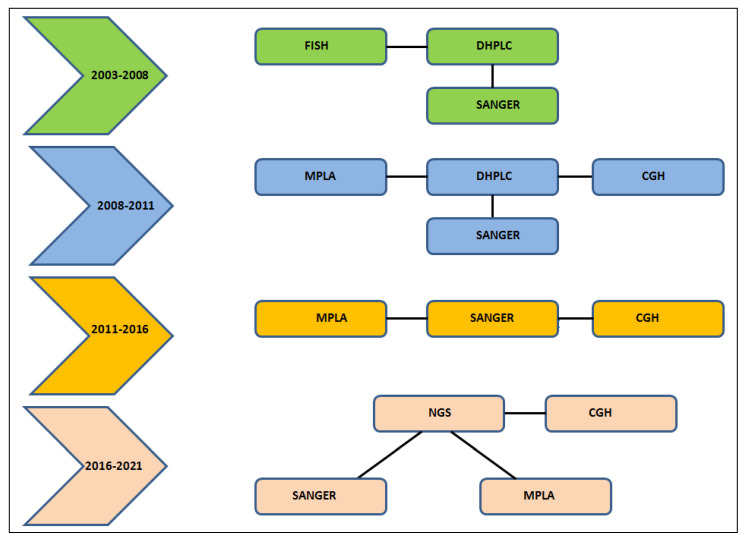
Workflow analysis. We reported the schematic representation of the whole study strategy used from 2003 to 2021. Several colors represent the different strategies of investigation in the 18 years of laboratory analysis.

Considering the long time period in which this activity was performed, we would like to specify the evolution of the diagnostic workflow over the last 18 years (Figure 1). Across the years, fluorescent in situ hybridization (FISH) was replaced by array-comparative genomic hybridization (a-CGH), denaturing high-performance liquid chromatography (DHPLC) by Sanger analysis. Since 2017 to date, next generation sequencing (NGS) is the first-tier test for variant identification in Sotos patients. Sanger sequencing and multiplex ligation-dependent probe amplification (MLPA) analysis are still used to confirm pathogenetic variants identified by the NGS approach.

### 2.3. Genomic DNA Extraction

Genomic DNA (gDNA) was obtained from peripheral blood cells using the DNA Mini Extraction Kit (Qiagen, Hilden, Germany), the EZ1 DNA Blood Kit on an EZ1 Advanced XL automatic extractor (QIAGEN GmbH, Germany), or QIA symphony S (Qiagen, Hilden, Germany) following the manufacturer’s instructions. DNA concentration was estimated by the spectrophotometric method for Sanger sequencing and MLPA and with the Qubit^®^ dsDNA HS Assay Kit (Thermo Fisher Foster City, CA, USA) on a Qubit^®^ 2.0 Fluorometer for NGS analysis.

### 2.4. Microdeletions and Intragenic Deletion Identification

The search for microdeletions and intragenic deletions was performed until 2017 with different technical approaches:

- FISH;
- MLPA;
- DHPLC;
- Direct Sanger sequencing.

#### 2.4.1. FISH

Since August 2008, the first step for the identification of microdeletions of the 5q35.3 region was represented by FISH analysis with PAC RP1-118M12 encompassing the *NSD1* gene [30]. In the presence of a deletion, FISH analysis was extended to probes RP11-147K7 and RP11-1006E8. FISH was performed as described by Lichter and Cremer (1992). Post-hybridization washing was performed in 0.1× SSC at 60 °C for 15 min and 4× SSC, Tween-20 0.1% at 42 °C for 15 min. Hybridization was detected by Avidina-Cy3 (Amersham Biosciences, Little Chalfont, Buckinghamshire, UK). Slides were counterstained with 4′,6-diamidino-2-phenylindole (DAPI) (200 ng/mL) and analyzed by fluorescence microscope Olympus BX70 equipped with a cooled CCD Video Camera Image Point, Photometrics; image analysis was carried out with PSI MacProbe software (Applied Imaging, Newcastle-Upon-Tyne, UK).

#### 2.4.2. MLPA

MLPA analysis, unlike FISH, recognizes both the deletions of the entire NSD1 gene and microdeletions of one or more exons.

The analysis was carried out on genomic DNA with the MLPA SALSA P026 Kit (MRC-Holland, Amsterdam, The Netherlands). All reactions (denaturation, ligation, and PCR) were performed following the manufacturer’s instructions. PCR products were run on a 3130xl automated sequencer (Applied Biosystems, Foster City, CA, USA) and data were analyzed using Genemapper v 3.2 and Coffalyser v.140721.1958 software (Applied Biosystems, Foster City). In selected cases, Array-CGH analysis was carried out to define the size and the breaking point of the deletions.

Array-CGH was performed using Superprint G3 CGH 8 × 60 K (Agilent Technologies, Santa Clara, CA, USA) according to the manufacturers’ protocol. Data were analyzed by Agilent Cytogenomics 4.0.3.12 software (Agilent Technologies, Santa Clara, CA, USA). All genomic positions were reported according to the human genome assembly (GRCh37/hg19).

#### 2.4.3. DHPLC

Eight hundred and fifteen patients were evaluated for intragenic mutations of NSD1 by DHPLC. The mutation analysis consisted of a first screening through DHPLC followed by the sequencing of only the fragments that showed a mobility shift.

The 22 coding exons and intron–exon boundaries were screened in 37 fragments. Exons longer than 470 bp were amplified using overlapping primer pairs. Aliquots of 50 ng of genomic DNA were amplified in a 25 mL reaction mix including 1× PCR Buffer (Invitrogen by Life Technologies Ltd., Paisley, UK), 1.5 mM MgCl2 (Invitrogen by Life Technologies Ltd., Paisley, UK), 200 mM dNTPs, 0.4 mM primers, and 0.5 U of Taq Platinum (Invitrogen by Life Technologies Ltd., Paisley, UK); all fragments were amplified using the following PCR conditions: 94 °C for 4 min, followed by 40 cycles at 94 °C for 30 min, 58–60 °C for 30 s, 72 °C for 30 s, and 72 °C for 7 min. Patients were screened through the 37 fragments by DHPLC on the WAVE Nucleic Acid Fragment Analysis System (Transgenomic); the DHPLC analysis was performed using from one to three temperatures per fragment.

#### 2.4.4. Sanger Sequencing

Four hundred and seventy-six patients were evaluated for intragenic mutations by Sanger sequencing. The sequencing was performed using the BigDye Terminator v3.1 Cycle Sequencing Kit (Applied Biosystems; Thermo Fisher Scientific, Inc.) and the ABI 3130xl and 3730 Automated Sequencers (Applied Biosystems, Foster City, CA, USA). The sequencing results were interpreted using Sequencing Analysis software updated to the last available version at the time of sequencing.

### 2.5. NGS

Since 2017, intragenic mutations and large genomic rearrangements (LGRs) have been identified by NGS using a Sophia Custom Panel (ID: COMS_2346) (SOPHIA Genetics, Saint-Sulpice, Switzerland).

The panel targets the entire coding region and the exon–intron boundaries (+/−5 bp) of the *NSD1* gene (NM_022455.4) and of another 29 genes associated with overgrowth: *AKT1* (NM_005163.5), *AKT2* (NM_001626.5), *AKT3* (NM_005465.4), *APC2* (NM_005883.2), *BRWD3* (NM_153252.4), *CCND2* (NM_001759.3), *CHD8* (NM_001170629.1), *DIS3L2* (NM_152383.4), *NMT3A* (NM_022552.4), *EED* (NM_001308007.1), *EZH2* (NM_004456.4), *GPC3* (NM_001164617.1), *GPC4* (NM_001448.2), *HERC1* (NM_003922.3), *HIST1H1E* (NM_005321.2), *IGF2* (NM_000612.5), *MTOR* (NM_004958.3), *NFIX* (NM_001271044.2), *PDK1* (NM_001278549.1), *PDK2* (NM_002611.4), *PIK3CA* (NM_006218.2), *PPP2R1A* (NM_006243.3), *PPP2R5D* (NM_006245.3), *PTCH1* (NM_000264.4), *PTCH2* (NM_003738.4), *PTEN* (NM_000314.8), *SETD2* (NM_014159.6), *SUFU* (NM_016169.3), *TCF20* (NM_005650.3).

The panel was validated on a total of 12 positive samples with known mutations ranging from single base substitutions to microdeletions to deletions of whole exons.

Through NGS, 239 patients with clinical suspicion of SoS or childhood overgrowth were evaluated.

#### 2.5.1. Library Preparation and NGS Sequencing

Two hundred nanograms of genomic DNA of patients was enriched using the Sophia Custom Panel according to the manufacturer’s instructions.

The capture-based target enrichment of 30 overgrowth related genes and the library construction protocols were carried out exclusively with the automated procedure implemented on the STARlet platform (Hamilton Company, Reno, NV, USA).

Library quantification was carried out with fluorometric quantitation using the Qubit dsDNA High Sensitivity Kit (ThermoFisher Scientific, Waltham, MA, USA).

The sequencing process was performed on the Illumina MiSeq system (Illumina Inc., San Diego, CA, USA).

In the routine of our medium-throughput laboratory, the number of samples per preparation was 24, which were run onto a 600-cycle format V3 flow-cell, sequenced via the Illumina MiSeq platform according to the Illumina and SOPHiA GENETICS protocols.

#### 2.5.2. NGS Data Analysis

The sequencing data were simultaneously processed for single nucleotide variants (SNVs), indels, and copy number variations (CNVs) using the SOPHiA DDM software (Sophia Genetics, Saint-Sulpice) updated with the last available version at the time of sequencing.

Sequencing reads were filtered for low-quality reads, trimmed for adapter sequences, and tagged as belonging to the specific patient according to the barcode.

Using the spectrum of the expected mutations in the training set, the parameters for variant calling were established to minimize the number of false-positive results and guarantee the characterization of all the true-positive calls. The following filter thresholds were considered: minimum allele frequency for single-nucleotide polymorphism (SNP) and indel (SNP% ≥ 20), phred-like quality score of the called variant (Qcall ≥ 20) and depth of coverage (Depth ≥ 20).

Variants were annotated according to nomenclature used by the Human Genome Variation Society (http://www.hgvs.org), accessed on 30 May 2022.

### 2.6. Variant Classification and Database Repository

All the detected sequence variations were submitted to the following databases: Human reference Genome GRCh37-hg19, Human Gene Mutation Database (HGMD), dbSNP151, ClinVar Database [31], Leiden Open Variation Database (LOVD) [32], Alamut (v.2.15), Varsome Database, and were searched for in the literature data.

The evaluation of the novel variants was based on the location, type, and evolutionary conservation of mutated amino acids, the biophysical and biochemical differences between wild type and mutant amino acids, and the in-silico analysis of the mutant sequence protein and inheritance pattern.

In silico analysis to predict the potential impact of the variants on the structure and function of protein was performed using the following tools: PolyPhen2 [33], SIFT, and Mutation Taster [34].

Starting from 2015, the variants identified were classified into five categories: pathogenic (5), likely pathogenic (4), variant of uncertain significance (3), likely benign (2), and benign (1), according to the guidelines provided by European Journal of Human Genetics [35] and interpreted using the guidelines provided by the American College of Medical Genetics and Genomics (ACMG) [36].

All of the novel variants detected by NGS and classified as pathogenic or likely pathogenic were confirmed by bidirectional Sanger sequencing. Novel *NSD1* variants have been deposited in the LOVD (https://grenada.lumc.nl/LOVD2/mendelian_genes/home.php?select_db=NSD1), accessed on 6 June 2020.

### 2.7. Reclassification of VUS Variants

Novel VUS out of 115 *NSD1* variants not reported in the literature were reviewed and reclassified according to their familial segregation, where available, and to the ACMG/AMP guidelines [36] based on the literature, public databases such as LOVD, VarSome [37], and ClinVar. At the end, the initial VUS interpretation and the new classification were compared. Statistical analysis was performed with GraphPad Prism software version 9.0. Categorical variables, given as a percentage of group totals, were analyzed through chi-square with rate correction. A *p*-value less than 0.05 was considered statistically significant.

## 3. Results

The molecular analysis allowed for the identification of 292 patients (281 with clinical suspicion of SoS and 11 with not-specific-overgrowth) with one *NSD1* variant out of 1530 patients analyzed, with a detection rate of 19.1% (N = 292/1530).

Over the years, the detection rate has changed according to the molecular method used.

▪ Two-hundred and sixty-nine patients were carriers of intragenic gene variations (17.6%; N = 269/1530);
▪ Thirteen individuals were carriers of 5q35 microdeletions encompassing the entire *NSD1* gene (0.9%; N = 13/1530);
▪ Ten were carriers of exon gene deletions (0.7%; N = 10/1530);
▪ One thousand two hundred and thirty eight were negative for the NSD1 variants (81%; N = 1238/1530).

An already described intragenic pathogenetic mutation was detected in 143 patients (52.1%; 143/269) and a variant of uncertain significance (VUS) in seven patients (2.6%; 7/269).

**Figure 2:**
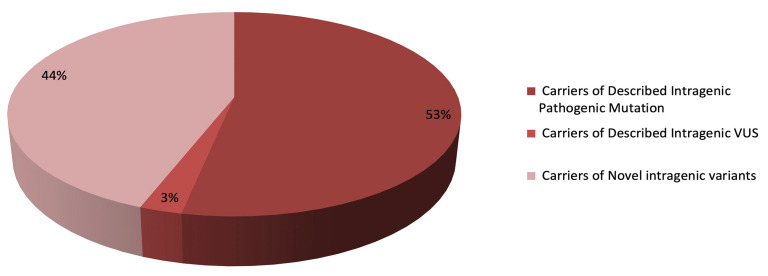
Percentage of carriers of intragenic variants. We show in dark red the patients carrying the described intragenic pathogenic mutation; in light red is shown the patients carrying the described intragenic VUS; in pink are patients carrying novel intragenic variants.

One hundred and nineteen patients were carriers of a intragenic variant never previously reported in the literature and in common databases (44.2%; 119/269) (Figure 2).

### 3.1. Intragenic Novel Variants of NSD1 Gene

The present study was particularly focused on 115 intragenic novel variants in the *NSD1* gene, never previously described in the literature and reported for the first time in 119 patients of the study here discussed (7.8%; N = 119/1530).

Among the new identified variants, 42% (*N* = 48/115) were insertions and/or deletions with frameshift consequences; 30% (*N* = 35/115) were missense; 15% (*N* = 17/115) were nonsense; 6% (*N* = 7/115) were splice site alterations; 3.5% (N = 4/115) were intronic; 1.7% (N = 2/115) were non-frameshift deletion; 1.7% (N = 2/115) were synonymous variants.

**Figure 3:**
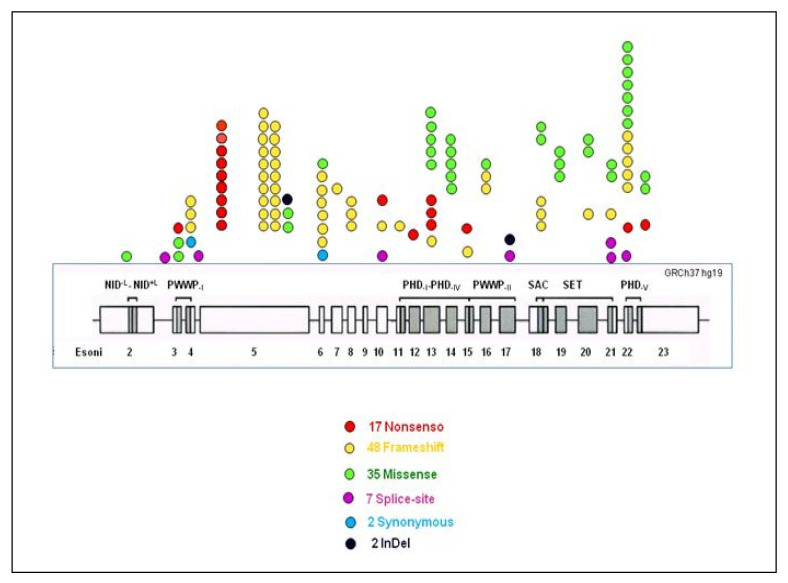
Schematic representation of the NSD1 protein domain and localization of 115 novel variants identified in this study. We show the color legend for the missense, nonsense, ins/dels, splicing, and intragenic deletions. Exons and introns are in scale and intronic variants are not reported.

From our data, variants were spread fairly evenly throughout the gene between exons 4 and 23; nevertheless, in exon 5, there was a cluster of truncating variations (43.07%; N = 28/65) and between exons 13 and 23 in the C-terminal half of NSD1, there were missense variations in highly conserved functional domains (77.1%; N = 27/35) (Figure 3). The above results are aligned with the data reported in Douglas et al. (2003) [7].

Segregation analysis was performed by the direct sequencing of parents when available.

In three cases (Sotos 916, Sotos 924, Sotos 526), we identified a familial variant, in particular two paternal inheritance and one maternal inheritance. We also identified a de novo variant in two monozygotic twins (Sotos 590 and Sotos 591).

Moreover, five variants (three frameshift, one missense, and one synonymous) were identified in more than one patient, in non-consanguineous families, as already reported in the literature for other variants [38].

The genotypic characteristics of the 119 patients in which a *NSD1* novel variant was identified are reported in Table S1 in the Supplementary Materials.

### 3.2. Reclassification of VUS in NSD1 Gene

Out of the 115 *NSD1* new variants identified, 32 variants were classified as VUS in the original records.

After genotyping the parents and re-analysis, 25/32 (78.1%) missense variants changed pathogenicity class: 5/32 (15.6%) were reclassified as likely benign because it was inherited from not affected parents and 20/32 (62.5%) as likely pathogenic because identified as de novo in proband. This shift toward classes was considered as statistically significant (*p* < 0.01). We found that only missense variants classified as VUS changed their classification across the years (Table 1). Finally, we observed that two variants (c.914A > G; c.947C > A), previously classified as VUS following the segregation analysis and re-interpretation, changed in class−2 because it was inherited from not affected parents.

### 3.3. Microdeletion of the 5q35 Region and Intragenic Deletion

Considering all the 1530 total patients with suspected Sos or overgrowth, we identified 13 subjects with 5q35 de novo microdeletions encompassing the entire *NSD1* gene.

In all patients, *NSD1* is present in a single copy (haploinsufficiency), showing a deletion of the gene in the heterozygous state.

For four patients with 5q35 microdeletion, FISH was the only analysis performed, while nine patients were analyzed by MLPA and array−CGH to precisely define the size of the deletions, as reported in Table 2.

The deletion size resulted in being very variable, ranging from 0.37 to 2.2 Mb as reported in the Japanese population [29,38].

In 10 patients, we identified nine different exonic deletions, as reported in Table 3.

The deletion of exons 2 and 3 was already described by Tatton Brown K et al. in 2005 [39].

The deletion of exon 15 was recurrent in two different patients (Sotos 530 and Sotos 1372) and was published in 2011 in the *India Academy of Sciences Journal* by Piccione M et al. [40].

### 3.4. Intragenic Variants Identified in NFIX, PTEN, EZH2, TCF20, BRWD3, and PPP2R5D Genes in Patients with Overgrowth

Since 2017, we have screened the patients by NGS on 29 genes implicated in overgrowth, in addition to *NSD1*. Nine patients out of 239, referred as overgrowth syndrome, were analyzed through NGS, which resulted in carrying a mutation in a different gene than *NSD1* (Table 4).

We identified five novel variants neither previously described in the literature nor reported in common databases and four mutations already present in the public database.

In detail, we detected two novel frameshift variants in the *NFIX* gene (c.664del; p.Val222Tyrfs*30 and c.1021del; p.His341Thrfs*52), one missense novel variant in the *EZH2* gene (c.449T > C; p.Ile150Thr), one novel nonsense in the BRWD3 gene (c.4252C > T; p.Arg1418*), and one novel nonsense in the TCF20 gene (c.3274C > T; p. Gln1092*).

Based on the ACMG criteria, these variants were classified as class 5-pathogenic or class 4-likely pathogenic variants.

Among the four already described mutations, the nonsense variant c.1003C > T; p.Arg335* in the *PTEN* gene was present in dbSNP (*rs121909231*) with an allelic frequency of 0.0007%, and in the HGMD-(*CM971278*- *Cowden 1 Syndrome*) and was described as class 4-likely pathogenic; the missense variant c.83T > C; p. Ile28Thr was in a functional domain, reported in the dbSNP database (*rs1355570425*) with an allelic frequency of 0.00040% and was classified as class 4-likely pathogenic. Two missense de novo variants (758G > A; p.Arg253Gln and c.1258G > A; p.Glu420Lys) in the *PPP2R5D* gene were present in the dbSNP database (respectively, *rs1131691266* with an allelic frequency of 0.0008%, and *rs863225080* with no frequency reported) and were classified as class 5-pathogenic. The c.1258G > A was also described in HGMD (*CM1513245-Disease Mutation-Intellectual Disability*).

## 4. Discussion

This work reports on the results, over the years, of the molecular analysis of the *NSD1* gene in a cohort of 1530 probands with clinical suspicion of SoS or overgrowth.

Briefly, out of the 1530 patients in our cohort, 815 were analyzed by DHPLC; 476 by Sanger sequencing; and 239 by NGS. This approach allowed for the identification of a *NSD1* variant in 292 patients, with a detection rate of 19.1%. Our detection rate was much lower than the one described in the literature (85%) by Tatton Brown [5].

It is important to emphasize that the selection of cases based on a good clinical diagnosis is crucial in determining the detection rate. For this reason, in a referral diagnostic laboratory, the detection rate is significantly lower (13%) [41] compared to that in strictly diagnosed patient groups (90–93%) [28,39].

Later on, the introduction of the NGS technique lowered the costs of diagnostic tests, allowing for the introduction of gene panels associated with overgrowth and intellectual disability (OGID), which, on one hand, have increased the sensitivity of the test, but have also lowered the stringency of the clinical criteria to undergo molecular testing, keeping the detection rate of *NSD1* still low (around 20%).

In order to facilitate and speed up the diagnosis, we underline the utility of sharing variant classification and we report 115 intragenic variants of the *NSD1* gene not yet described.

However, this implies a continuous effort to establish a direct communication between the laboratory and the referring physician.

The mutational spectrum observed in our study mainly consisted of single nucleotide variants spread fairly evenly throughout the gene. Nevertheless, a clustering of truncating mutations in exon 5 (41.5%) and of missense variants in highly conserved functional domains between exons 13 and 23 of the *NSD1* protein (77%) were present. Half of the identified variants are protein truncation variants (PTVs) (56.5%) and 30% are missense, of which a third involves a cysteine residue that plays an important role in correct protein refolding [42].

Overall, only six variants were recurrent in more than one patient, further confirming the almost “private” nature of the *NSD1* mutations.

Out of the 115 *NSD1* new variants identified, the laboratory records showed the presence of 32 VUS. After genotyping the parents and after the re-analysis, 25/32 VUS changed pathogenicity class and were re-classified and re-interpreted as class 2 or class 4, while seven of them did not change the classification level.

The identification of VUS is common and remains a challenge in clinical practice, mostly because of the possibility of changing into the phatogenic class, as reported in 62.5% of our cases (20/32). These data confirm the importance of periodical reinterpretation of VUS with the intent of recontacting patients and their physician to inform them during a follow-up.

For this scope, we underline the utility of sharing variants to facilitate reclassification and possible re-interpretation to also speed up the diagnosis in other centers, with a continuous effort to establish a direct communication between the laboratory and the referring physician.

The reclassification may have a potential crucial impact on families, ensuring a deeper understanding of the outcome of genetic testing. For this reason, the possibility that a variant can be reclassified over time should be raised with patients during pre-test counseling and consent.

Our results, in agreement with other European studies, confirm that the intragenic point mutations of the *NSD1* gene are the main cause of SoS in Western patients and that the condition appears as a haploinsufficiency syndrome [7,24,25,26,27,28].

About 9% of individuals with a SoS of European ancestry and 50% of Japanese ancestry have a specific deletion of the chromosomal region flanking the *NSD1* gene [43].

In our series of patients, the deletions of the whole gene occurred in only 1% (13 subjects) of patients with suspected SoS, confirming that the microdeletions are rare in non-Japanese patients.

Partial *NSD1* deletions were also present in 10 individuals, representing only 1% of our subjects, a slightly lower value than the 5% reported in the literature [44].

SoS usually arises from *NSD1* de novo mutations in the affected subject, as also confirmed by 26.9% (32/119) of the cases in the present study. Less than 2% of subjects in familial cases (2.6% in our clinical cases) with autosomal dominant transmission have been reported [45].

It has been suggested that this lack of family cases could be related to the presence of intellectual disability and a underlying defect in fertility associated with *NSD1* mutations, which can affect the possibility of having offspring [22].

Furthermore, the literature data demonstrate that familial cases generally present missense mutations.

In the present study, two familial cases were caused by missense mutations and only one by a splice site mutation.

Sotos syndrome is the most common syndrome within the overgrowth with intellectual disability (OGID) category [21]. However, a sharp differential diagnosis with other syndromes in this category including Weaver syndrome (OMIM*277590), Bannayan–Riley–Ruvalcaba syndrome (OMIM 138350)**,** Malan syndrome (OMIM #614753), and *BRWD3*-related disorder (MIM: 300553) is mandatory.

The introduction of NGS in the molecular diagnosis of human overgrowth syndromes allowed us to identify nine intragenic novel mutations in six different genes associated with OGID and to explain about 4% (9/239) of cases of this cohort (analyzed with NGS), which resulted in being negative for mutations in the *NSD1* gene. To note, eight of these nine patients had been referred with clinical suspicion of overgrowth and not as suspected Sotos.

In particular, we identified two novel mutations in the *NFIX* gene on chromosome 19p13 in two patients. *NFIX* encodes nuclear factor I/X and was reported as a causative gene for Sotos-like phenotypes (known as Sotos syndrome 2 or Malan syndrome (OMIM #614753) [46]. Additionally, mutations in this gene can cause Marshall–Smith syndrome (OMIM 602535), a syndrome of advanced bone age and increase length at birth. Malan and Marshall–Smith are two syndromes that present a different phenotype, with mutation in the same *NFIX* gene [4,47].

Moreover, we observed in one patient with clinical suspicion of SoS and in one with overgrowth, two novel mutations in *PTEN*. This gene is a tumor suppressor that is implicated in the phosphoinositol 3-kinase (PI3K/AKT) pathway, and is involved in the regulation of growth, associated with Cowden-1 syndrome (OMIM 15835) and Bannayan–Riley–Ruvalcaba syndrome (OMIM 15348) [48].

These two syndromes are characterized by multiple hamartomas and have many overlapping features with SoS and overgrowth such as learning difficulties, macrocephaly, and tall stature.

Furthermore, we identified, in a patient with clinical suspicion of Weaver syndrome, a heterozygous de novo mutation in the enhancer of zeste homolog 2 (*EZH2*) gene, a histone methyltransferase responsible for histone H3 at lysine 27 (*H3K27*) trimethylation.

This result is consistent with studies showing de novo germline heterozygous mutations in *EZH2* in Weaver syndrome [49,50].

We identified two novel mutations, c.4252C > T (p.Arg1418*) in Bromodomain And WD Repeat Domain Containing 3 (*BRWD3)* and c.3274C > T (p.Gln1092*) in transcription factor 20 (*TCF20)* in patients with suspected overgrowth. The *BRWD3* gene (MIM 300553), located at Xq21.1, is associated with X-linked mental retardation and macrocephaly, while *TCF20* (MIM *603107) variants with intellectual disability and postnatal overgrowth [51].

The use of the panel of genes associated with OGID therefore allowed us to formulate a differential molecular diagnosis among the overlapping phenotypes in nine patients in our cohort.

Thus, considering the significant phenotypic variability and the importance of an adequate early multidisciplinary therapeutic program, the diagnosis of SoS inevitably requires molecular confirmation.

Confirmation of a diagnosis is extremely important for the psychosocial health of a family, and it also signifies the end of the stressful search for diagnosis with different, often invasive, methods. The natural history of SoS is fairly well-known and recommendations for follow-up exist, which are important issues in the counseling of the family [52].

In the future, it will be important to arrange consensus conferences to bring together international experts with the aim to standardize the clinical criteria for the diagnosis of SoS and to better define epidemiology, pathogenesis, and management of the disease.

We believe that efforts toward the creation of international registers of SoS are needed, as recently carried out for other overgrowth syndromes such as Malan syndrome (https://www.malansyndrome.org/global-patient-registry (accessed on 6 June 2020)) to improve the clinical, epidemiological, etiopathogenic, and natural history knowledge of the disease.

In light of this, the continuous update and reports of variants detected in Sotos patients, are crucial to improve the diagnostic criteria of the guidelines.

In conclusion, our study reports the results of 18 years of diagnostic activity in different workflow settings, underlying the utility of sharing the variant classification and possible re-interpretation to facilitate and speed up the diagnosis in other centers. However, this implies a continuous effort to establish a direct communication between the laboratory and the referring physician.

## Figures and Tables

**Table 1:** Missense VUS reclassification.

Unchanged N/T (%)	Reclassified N/T (%)	*p*-Value (Chi-Square)	New Class−2	New Class−4	Unchanged
7/32 (21.8%)	25/32 (78.1%)	<0.01	5	20	7

**Table 2:** Chromosomal arrangement in SoS patients.

Case	Chromosomal Arrangement Size	Locations	Inheritance
Sotos 764	357 Kb	5q35.2 (176,378,453–176,735,244)del	De novo
Sotos 780	1.8 Mb	5q35.2 (175,576,602–177,422,760)del	De novo
Sotos 840	1.8 Mb	5q35.2 (175,509,208–177,355,366)del	De novo
Sotos 646	2.2 Mb	5q35.3 (175,243,487–177,501,801)del	De novo
Sotos 753	1.1 Mb	5q35.3 (175,719,197–176,883,275)del	De novo
Sotos 848	1.98 Mb	5q35.2 (175,437,847–177,422,760)del	De novo
Sotos 703	1.3 Mb	5q35.3 (175,509,208–176,837,404)del	De novo
Sotos 859	2.2 Mb	5q35.3 (175,347,741–177,587,471)del	De novo
Sotos 912	1.8 Mb	5q35.2q35.3 (175,576,602–177,422,760)del	De novo

**Table 3:** Intragenic deletions.

Intragenic Deletion (hg19)	Case	Bibliography
deletion exon 4	Sotos 1285	-
deletion 5′UTR to exon 15	Sotos 800	-
deletion 5′UTR to exon 3	Sotos 1236	-
deletion exon 2–3	Sotos 122	[38]
deletion exon 5–15	Sotos 118	-
deletion exon 7 and exon 18	Sotos 432	-
deletion exon 11 to exon 14	Sotos 658	-
deletion exon 15	Sotos 530/Sotos 1372	[39]
deletion exon 20	Sotos 1003	-

**Table 4:** Nine pathogenic or likely pathogenic variants identified in other genes in the present study.

Case	Clinical Suspicion	NM	Gene	Nucleotide Change	Protein Change	Location hg19	Type of Mutation	Inheritance	Protein Domain	Varsome/ACMG Criteria
OG008	OG	NM_001271043.2	*NFIX*	c.664del	p.Val222Tyrfs*30	ex 4	FS	de novo	/	Pathogenic (PVS1−PM2-PP3)
19-MOG-0052	OG	NM_001271043.2	*NFIX*	c.1021del	p.His341Thrfs*52	ex 7	FS	de novo	/	Pathogenic (PVS1−PM2-PP3)
20-MOG-0048	sWS	NM_004456.4	*EZH2*	c.449T > C	p.Ile150Thr	ex 5	MS	de novo	/	Likely Pathogenic (PM2−PM1-PP2-PP3)
OG30	OG	NM_153252.4	*BRWD3*	c.4252C > T	p.Arg1418*	ex 38	NS	n.p.	Bromodomain	Pathogenic (PVS1−PM2-PP3)
19-MOG-0041	OG	NM_005650.3	*TCF20*	c.3274C > T	p.Gln1092*	ex 1	NS	de novo	/	Pathogenic (PVS1−PM2-PP3)
20-MOG-0002	sSoS	NM_000314.8	*PTEN*	c.83T > C	p.Ile28Thr	ex 2	MS	n.p.	PTP	Likely pathogenic (PM1−PM2-PP2−PP3)
20-MOG-0021	OG	NM_000314.8	*PTEN*	c.1003C > T	p.Arg335*	ex 8	NS	n.p.	/	Pathogenic (PS3−PVS1−PP5−PM2−PP3)
OG41	OG	NM_006245.3	*PPP2R5D*	c.758G > A	p.Arg253Gln	ex 7	MS	de novo	B56	Pathogenic (PM2−PM5−PM1-PP2-PP3)
21-MOG-0010	OG	NM_006245.3	*PPP2R5D*	c.1258G > A	p.Glu420Lys	ex 12	MS	de novo	B56	Pathogenic (PP5-PM2-PP2-PP3)

Abbreviations: n.p. = not performed; ex = exon; MS = missense; NS = nonsense; FS = frameshift; sSoS = suspected Sotos syndrome; sWS = suspected Weaver syndrome; OG = overgrowth.

## Data Availability

Novel *NSD1* variants have been deposited in the LOVD (https://grenada.lumc.nl/LOVD2/mendelian_genes/home.php?select_db=NSD1, ID: NSD1_000319, NSD1_000370, NSD1_000366, NSD1_000281, NSD1_000368, NSD1_00302567, NSD1_00302571, NSD1_00302572, NSD1_00302573, NSD1_00302574, NSD1_00302679, NSD1_00302843, NSD1_00302844, NSD1_00302845, NSD1_00302846, NSD1_00302847, NSD1_00302848, NSD1_00302850, NSD1_00302851, NSD1_00302852, NSD1_00302855, NSD1_00302856, NSD1_00302857, NSD1_00302858, NSD1_00302859, NSD1_00302860, NSD1_00302861, NSD1_00302862, NSD1_00302863, NSD1_00302864, NSD1_00302865, NSD1_00302867, NSD1_00302868, NSD1_00302869, NSD1_00303567, NSD1_00303569, NSD1_00303570, NSD1_00303571, NSD1_00303572, NSD1_00303573, NSD1_00303574, NSD1_00303575, NSD1_00303576, NSD1_00303577, NSD1_00303578, NSD1_00303579, NSD1_00303580, NSD1_00303581, NSD1_00303582, NSD1_00303583, NSD1_00303584, NSD1_00303607, NSD1_00303608, NSD1_00303609, NSD1_00303610, NSD1_00303611, NSD1_00303612, NSD1_00303613, NSD1_00303614, NSD1_00303615, NSD1_00303616, NSD1_00303617, NSD1_00303082, NSD1_00303083, NSD1_00302823, NSD1_00303084, NSD1_00303085, NSD1_00303086, NSD1_00303091, NSD1_00303092, NSD1_00303363, NSD1_00303365, NSD1_00303366, NSD1_00303966, NSD1_00303368, NSD1_00303367, NSD1_00302834, NSD1_00303522, NSD1_00303523, NSD1_00303524, NSD1_00303525, NSD1_00303526, NSD1_00303967, NSD1_00303968, NSD1_00303969, NSD1_00303970, NSD1_00303971, NSD1_00303972, NSD1_00303973, NSD1_00303974, NSD1_00303975, NSD1_00303978, NSD1_00303979, NSD1_00303980, NSD1_00303981, NSD1_00303982, NSD1_00303983, NSD1_00303984, NSD1_00303985, NSD1_00303986, NSD1_00303987, NSD1_00303988, NSD1_00303989, NSD1_00303990, NSD1_00303991, NSD1_00303992, NSD1_00303993, NSD1_00303994, NSD1_00303996, NSD1_00303995).

## Supplementary Materials

The following supporting information can be downloaded at: https://www.mdpi.com/article/10.3390/genes14020295/s1, Table S1: One hundred and fifteen *NSD1* variants were identified in the present study.
