# Supplementary material for: Molecular Analysis and Reclassification of NSD1 Gene Variants in a Cohort of Patients with Clinical Suspicion of Sotos Syndrome
Source: Genes (Basel). 2023 Jan 22;14(2):295. doi: 10.3390/genes14020295 (PMC9956575; doi:10.3390/genes14020295)
Supplement: Supplementary file 1 [file genes-14-00295-s001.zip › genes-2134420-supplementary.pdf]

**Table S1. One hundred and fifteen NSD1 variants were identified in the present study.**

| Case | Clinical suspicion | Nucleotide change | Protein change | Location hg19 | Type of mutation | Inherit-ance | N. of carriers | Protein Domain | Varsome / ACMG criteria |
|---|---|---|---|---|---|---|---|---|---|
| 913 | sSoS | c.1007G>A | p.Trp336* | ex3 | NS | n.p. | 1 | PWWP1 | Pathogenic (PVS1-PM2-PP3) |
| 1030 | sSoS | c.1482C>A | p.Cys494* | ex5 | NS | n.p. | 1 | / | Pathogenic (PVS1-PM2-PP3) |
| 116 | sSoS | c.2151_2152insTA | p.Ser718* | ex5 | NS | n.p. | 1 | / | Likely Pathogenic (PVS1-PM2) |
| **1363** | sSoS | c.2333T>A | p.Leu778* | ex5 | NS | n.p. | 1 | / | Pathogenic (PVS1-PM2-PP3) |
| 773 | sSoS | c.2435del | p.Leu812* | ex5 | NS | n.p. | 1 | / | LikelyPathogenic (PVS1-PM2) |
| 656 | sSoS | c.2587G>T | p.Glu863* | ex5 | NS | n.p. | 1 | / | Pathogenic (PVS1-PM2-PP3) |
| 742 | sSoS | c.3007A>T | p.Arg1003* | ex5 | NS | n.p. | 1 | / | Pathogenic (PVS1-PM2-PP3) |
| 217 | sSoS | c.3028A>T | p.Lys1010* | ex5 | NS | n.p. | 1 | / | Pathogenic (PVS1-PM2-PP3) |
| 430 | sSoS | c.3629delC | p.Ser1210* | ex5 | NS | n.p. | 1 | / | Likely Pathogenic (PVS1-PM2) |
| 1280 | sSoS | c.3767_3768insAT | p.Thr1257* | ex5 | NS | de novo | 1 | / | Pathogenic (PVS1-PM2-PP3) |
| 874 | sSoS | c.4660G>T | p.Glu1554* | ex12 | NS | n.p. | 1 | PHD1 | Pathogenic (PVS1-PM2-PP3) |
| 958 | sSoS | c.4792C>T | p.Gln1598* | ex13 | NS | de novo | 1 | PHD2 | Pathogenic (PVS1-PM2-PP3) |
| 349 | sSoS | c.4810A>T | p.Lys1604* | ex13 | NS | n.p. | 1 | PHD2 | Pathogenic (PVS1-PM2-PP3) |
| 466 | sSoS | c.4869C>A | p.Tyr1623* | ex13 | NS | n.p. | 1 | PHD2 | Pathogenic (PVS1-PM2-PP3) |
| 600 | sSoS | c.5166_5167del | p.Cys1722* | ex15 | NS | n.p. | 1 | PHD4 | Pathogenic (PVS1-PM2-PP3) |
| OG10 | Overgrowth | c.6316A>T | p.Lys2106* | ex22 | NS | n.p. | 1 | / | Pathogenic (PVS1-PM2-PP3) |
| 21-MOG-008 | sSoS | c.6501C>A | p.Cys2167* | ex23 | NS | n.p. | 1 | C5HCH | Pathogenic (PVS1-PM2-PP3) |
| 126 | / | c.4954dup | p.Ser1652Phefs*4 | ex13 | FS | n.p. | 1 | PHD3 | Pathogenic (PVS1-PM2-PP3) |
| 631 | sSoS | c.1086_1089del | p.Gln363Thrfs*55 | ex4 | FS | n.p. | 1 | PWWP1 | Pathogenic (PVS1-PM2-PP3) |
| 881 | sSoS | c.1107del(T) | p.Phe369Leufs*50 | ex4 | FS | de novo | 1 | PWWP1 | Pathogenic (PVS1-PM2-PP3) |
| 398 | sSoS | c.1222_1223insAA | p.Gly408Glufs*12 | ex4 | FS | n.p. | 1 | PWWP1 | Pathogenic (PVS1-PM2-PP3) |
| 313 | sSoS | c.1447del(T) | p.Ser483Leufs*29 | ex5 | FS | n.p. | 1 | / | Pathogenic (PVS1-PM2-PP3) |
| 734 | sSoS | c.1527delT | p.Ser510Valfs*2 | ex5 | FS | de novo | 1 | / | Likely Pathogenic (PVS1-PM2) |

| | | | | | | | | | |
|---|---|---|---|---|---|---|---|---|---|
| 1108 | sSoS | c.1576del | p.Arg526Glyfs*10 | ex5 | FS | n.p. | 1 | / | Likely Pathogenic (PVS1-PM2) |
| 443 | sSoS | c.1633delA | p.Thr545Argfs*10 | ex5 | FS | n.p. | 1 | / | Pathogenic (PVS1-PM2-PP3) |
| 242 | sSoS | c.1662_1663delinsC | p.Ala555Glnfs*44 | ex5 | FS | n.p. | 1 | / | Pathogenic (PVS1-PM2-PP3) |
| 1182 | sSoS | c.1667_1685del | p.Asn556Thrfs*37 | ex6 | FS | n.p. | 1 | / | Pathogenic (PVS1-PM2-PP3) |
| 1194 | sSoS | c.1981_1982del | p.Ser661Cysfs*3 | ex6 | FS | de novo | 1 | / | Pathogenic (PVS1-PM2-PP3) |
| 485, 259 | sSoS/sSoS | c.2258_2259insTC | p.Lys754Glnfs*15 | ex5 | FS | de novo/de novo | 2 | / | Likely Pathogenic (PVS1-PM2) |
| 130 | sSoS | c.2262_2263del | p.Lys754Asnfs*10 | ex5 | FS | n.p. | 1 | / | Likely Pathogenic (PVS1-PM2) |
| 1158 | sSoS | c.2482dup | p.Ser828Phefs*2 | ex6 | FS | de novo | 1 | / | Likely Pathogenic (PVS1-PM2) |
| 994 | sSoS | c.2487_2488del | p.Ser830Trpfs*14 | ex5 | FS | de novo | 1 | / | Pathogenic (PVS1-PM2-PP3) |
| 464 | Overgrowth | c.2648dup | p.Pro884Alafs*12 | ex5 | FS | n.p. | 1 | / | Pathogenic (PVS1-PM2-PP3) |
| 262 | / | c.2688_2689del | p.Asn897Serfs*5 | ex5 | FS | n.p. | 1 | / | Pathogenic (PVS1-PM2-PP3) |
| 776 | sSoS | c.2701dup | p.Ile901Asnfs*2 | ex5 | FS | de novo | 1 | / | Pathogenic (PVS1-PM2-PP3) |
| 1007 | sSoS | c.2833_2834insA | p.Ser945Tyrfs*3 | ex5 | FS | n.p. | 1 | / | Pathogenic (PVS1-PM2-PP3) |
| 579 | sSoS | c.2906delG | p.Gly969Glufs*71 | ex5 | FS | n.p. | 1 | / | Pathogenic (PVS1-PM2-PP3) |
| 322 | sSoS | c.3074dup | p.Pro1026Alafs*21 | ex5 | FS | n.p. | 1 | / | Pathogenic (PVS1-PM2-PP3) |
| 1002 | sSoS | c.3344_3348del | p.Lys1115Ilefs*2 | ex5 | FS | n.p. | 1 | / | Pathogenic (PVS1-PM2-PP3) |
| 1277 | sSoS | c.3344del | p.Lys1115Serfs*26 | ex5 | FS | de novo | 1 | / | Pathogenic (PVS1-PM2-PP3) |
| 457 | sSoS | c.3585delT | p.Val1196Cysfs*23 | ex5 | FS | n.p. | 1 | / | Likely Pathogenic (PVS1-PM2) |
| 1067 | sSoS | c.3656_3657del | p.Thr1219Argfs*6 | ex5 | FS | n.p. | 1 | / | Likely Pathogenic (PVS1-PM2) |
| 522 | sSoS | c.3778delG | p.Ala1260Leufs*49 | ex5 | FS | n.p. | 1 | / | Pathogenic (PVS1-PM2-PP3) |
| 630 | sSoS | c.3820delC | p.Arg1275Glyfs*34 | ex6 | FS | n.p. | 1 | / | Pathogenic (PVS1-PM2-PP3) |
| 245 | sSoS | c.3910dup | p.Gln1304Profs*10 | ex6 | FS | n.p. | 1 | / | Pathogenic (PVS1-PM2-PP3) |
| 588 | sSoS | c.3965dup | p.Ser1323Ilefs*2 | ex7 | FS | n.p. | 1 | / | Pathogenic (PVS1-PM2-PP3) |
| 975 | sSoS | c.4204del | p.Ser1402Valfs*17 | ex8 | FS | n.p. | 1 | / | Pathogenic (PVS1-PM2-PP3) |
| 796 | sSoS | c.4210-4211insTT | p.Arg1404Leufs*16 | ex8 | FS | n.p. | 1 | / | Pathogenic (PVS1-PM2-PP3) |

| | | | | | | | | | |
|---|---|---|---|---|---|---|---|---|---|
| 428 | sSoS | c.4277_4278delinsT | p.Lys1426Metfs*20 | ex8 | FS | n.p. | 1 | / | Pathogenic (PVS1-PM2-PP3) |
| 576 | sSoS | c.4281_4282dup | p.Asp1428Glyfs*19 | ex8 | FS | n.p. | 1 | / | Pathogenic (PVS1-PM2-PP3) |
| 208, 704 | sSoS/ sSoS | c.4455dup | p.Val1486Serfs*4 | ex10 | FS | de novo | 2 | / | Pathogenic (PVS1-PM2-PP3) |
| 473 | sSoS | c.4572delG | p.Met1525Cysfs*49 | ex11 | FS | n.p. | 1 | / | Pathogenic (PVS1-PM2-PP3) |
| 1305, 1356 | sSoS/sSoS | c.5019_5020del | p.Phe1673Leufs*12 | ex15 | FS | de novo | 2 | PHD3 | Pathogenic (PVS1-PM2-PP3) |
| 358 | sSoS | c.5356_5360del | p.Lys1786Glufs*3 | ex16 | FS | n.p. | 1 | PWWP2 | Pathogenic (PVS1-PM2-PP3) |
| OG25 | Overgrowth | c.5370_5385del | p.Asp1790Glufs*26 | ex16 | FS | de novo | 1 | PWWP2 | Pathogenic (PVS1-PM2-PP3) |
| 626 | sSoS | c.5644_5655delinsTGTA | p.Val1882Cysfs*7 | ex18 | FS | n.p. | 1 | / | Pathogenic (PVS1-PM2-PP3) |
| 536 | sSoS | c.5654delT | p.Phe1885Serfs*23 | ex18 | FS | n.p. | 1 | / | Pathogenic (PVS1-PM2-PP3) |
| 195 | Overgrowth | c.5728_5729dup | p.Cys1911Asnfs*59 | ex18 | FS | n.p. | 1 | SAC | Pathogenic (PVS1-PM2-PP3) |
| 797 | sSoS | c.6047_6050dup | p.Phe2018Serfs*12 | ex20 | FS | n.p. | 1 | SET | Pathogenic (PVS1-PM2-PP3) |
| 1074 | sSoS | c.6184del | p.Cys2062Valfs*22 | ex21 | FS | n.p. | 1 | SET | Pathogenic (PVS1-PM2-PP3) |
| 705 | sSoS | c.6303_6306del | p.Lys2101Asnfs*48 | ex22 | FS | n.p. | 1 | / | Pathogenic (PVS1-PM2-PP3) |
| 1113 | sSoS | c.6313_6314del | p.Gly2105Lysfs*7 | ex22 | FS | de novo | 1 | / | Pathogenic (PVS1-PM2-PP3) |
| 832 | sSoS | c.6358del | p.Glu2120Serfs*30 | ex22 | FS | n.p. | 1 | PHD5 | Pathogenic (PVS1-PM2-PP3) |
| 936 | sSoS | c.6452_6459del | p.Lys2151Serfs*12 | ex22 | FS | n.p. | 1 | PHD5 | Pathogenic (PVS1-PM2-PP3) |
| 354 | sSoS | c.6461del | p.Ala2154Glufs*140 | ex22 | FS | n.p. | 1 | PHD5 | Pathogenic (PVS1-PM2-PP3) |
| 681 † | sSoS | c.1601G>T | p.Gly534Val | ex5 | MS | n.p. | 1 | / | Likely Benign (M2-PP2-BP4) |
| 1333 † | sSoS | c.3676T>C | p.Cys1226Arg | ex6 | MS-Cys | n.p. | 1 | / | Likely Benign(PM2-PP2-BP4) |
| **1363** † | sSoS | c.3728G>C | p.Ser1243Thr | ex5 | MS | n.p. | 1 | / | Likely Benign(PM2-PP2-BP4) |
| 1229 † | sSoS | c.4672T>C | p.Cys1558Arg | ex13 | MS-Cys | n.p. | 1 | PHD1 | Likely Pathogenic (PM1-PM2-PP2-PP3) |
| 101 | sSoS | c.4786T>G | p.Cys1596Gly | ex13 | MS-Cys | n.p. | 1 | PHD2 | Likely Pathogenic (PM1-PM2-PM5-PP2-PP3) |
| 278 † | sSoS | c.4832G>A | p.Cys1611Tyr | ex13 | MS-Cys | n.p. | 1 | PHD2 | Likely Pathogenic (PM1-PM2-PP2-PP3) |

| | | | | | | | | | |
|---|---|---|---|---|---|---|---|---|---|
| 843 † | sSoS | c.4859T>A | p.Val1620Asp | ex13 | MS | de novo | 1 | PHD2 | Likely Pathogenic (PM1-PM2-PP2-PP3) |
| **924***  | sSoS | c.4883T>G | p.Met1628Arg | ex13 | MS | paternal | 1 | PHD2 | Vus (PM1-PM2-PP2-BP4) |
| **518 †** | sSoS | c.4982G>T | p.Cys1661Phe | ex14 | MS-Cys | n.p. | 1 | PHD3 | Likely Pathogenic (PM1-PM2-PP2-PP3) |
| **518 †** | sSoS | c.4990T>C | p.Cys1664Arg | ex14 | MS-Cys | n.p. | 1 | PHD3 | Likely Pathogenic (PM1-PM2-PP2-PP3) |
| 685 † | sSoS | c.5032G>T | p.Gly1678Trp | ex14 | MS | de novo | 1 | PHD3 | Likely Pathogenic (PM1-PM2-PP2-PP3) |
| 506 † | sSoS | c.5074C>A | p.His1692Asn | ex14 | MS | n.p. | 1 | PHD3 | Likely Pathogenic (PM1-PM2-PP2-PP3) |
| 1243 † | sSoS | c.5146G>A | p.Gly1716Arg | ex14 | MS | de novo | 1 | PHD4 | Likely Pathogenic (PM1-PM2-PP2-PP3) |
| 1184 | sSoS | c.521T>A | p.Val174Asp | ex3 | MS | n.p. | 1 | / | Likely Benign(PM2-PP2) |
| 736 † | sSoS | c.5311C>G | p.Pro1771Ala | ex16 | MS | n.p. | 1 | PWWP2 | Likely Pathogenic (PM1-PM2-PP2-PP3) |
| 154 | sSoS | c.5774G>C | p.Cys1925Ser | ex18 | MS-Cys | n.p. | 1 | SAC | Likely Pathogenic (PM1-PM2-PM5-PP2-PP3) |
| 979 † | Overgrowth | c.5867T>A | p.Leu1956Gln | ex18 | MS | de novo | 1 | SET | Likely Pathogenic (PM1-PM2-PP2-PP3) |
| 526* | sSoS | c.5899T>C | p.Phe1967Leu | ex19 | MS | maternal | 1 | SET | Likely Pathogenic (PM1-PM2-PP2-PP3) |
| 187 † | sSoS | c.5911T>A | p.Tyr1971Asn | ex19 | MS | n.p. | 1 | SET | Likely Pathogenic (PM1-PM2-PP2-PP3) |
| 98 | / | c.5995C>G | p.Leu1999Val | ex19 | MS | n.p. | 1 | SET | Likely Pathogenic (PM1-PM2-PM5-PP2-PP3) |
| 505 † | sSoS | c.6023A>G | p.Asp2008Gly | ex20 | MS | n.p. | 1 | SET | Likely Pathogenic (PM1-PM2-PP2-PP3) |
| 482 † | sSoS | c.6037G>A | p.Gly2013Arg | ex20 | MS | de novo | 1 | SET | Likely Pathogenic (PM1-PM2-PP2-PP3) |
| 660 † | sSoS/sWS | c.6178C>G | p.Leu2060Val | ex21 | MS | n.p. | 1 | SET | Likely Pathogenic (PM1-PM2-PP2-PP3) |
| **924 †** | sSoS | c.6179T>G | p.Leu2060Arg | ex21 | MS | n.p. | 1 | SET | Likely Pathogenic (PM1-PM2-PP2-PP3) |

| | | | | | | | | | |
|---|---|---|---|---|---|---|---|---|---|
| 1113 | sSoS | c.6308A>G | p.Gln2103Arg | ex22 | MS | de novo | 1 | / | Likely Benign(PM2-PP2-BP4) |
| 1056 † | sSoS | c.6350G>T | p.Arg2117Leu | ex22 | MS | n.p. | 1 | / | Vus (PM2-PP2-PP3) |
| 890 † | sSoS | c.6372T>G | p.Cys2124Trp | ex22 | MS-Cys | n.p. | 1 | PHD5 | Likely Pathogenic (PM1-PM2-PP2-PP3) |
| 916* † | sSoS | c.6392T>A | p.Val2131Asp | ex22 | MS | paternal | 1 | PHD5 | Likely Pathogenic (PM1-PM2-PP2-PP3) |
| 532 † | sSoS | c.6436T>G | p.Cys2146Gly | ex22 | MS-Cys | n.p. | 1 | PHD5 | Likely Pathogenic (PM1-PM2-PM5-PP2-PP3) |
| 84 † | / | c.6438T>G | p.Cys2146Trp | ex22 | MS-Cys | n.p. | 1 | PHD5 | Likely Pathogenic (PM1-PM2-PM5-PP2-PP3) |
| 978 † | Overgrowth | c.6458C>A | p.Pro2153Gln | ex22 | MS | de novo | 1 | PHD5 | Likely Pathogenic (PM1-PM2-PP2-PP3) |
| 1286 † | sSoS | c.6532T>C | p.Cys2178Arg | ex23 | MS-Cys | n.p. | 1 | C5HCH | Likely Pathogenic (PM1-PM2-PM5-PP2-PP3) |
| 623, 725 | sSoS/sSoS | c.6541_6542delTCinsAA | p.Ser2181Asn | ex23 | MS | de novo/ de novo | 2 | C5HCH | Likely Pathogenic (PM1-PM2-PM5-PP2-PP3) |
| 1117 † | sSoS | c.914A>G | p.Gln305Arg | ex2 | MS | maternal | 1 | / | Likely Benign(PM2-PP2) |
| 1004 † | sSoS | c.947C>A | p.Ser316Tyr | ex3 | MS | paternal | 1 | / | Likely Benign(PM2-PP2-PP3) |
| 590,591* | sSoS/ sSoS | c.1236+2T>G | / | int4 | SPL | de novo | 2 | / | Pathogenic (PVS1-PM2-PP3) |
| 1275 † | sSoS | c.4378+4_4378+5del | / | int10 | SPL | n.p. | 1 | / | Vus(PM2-PP3) |
| 805 | sSoS | c.5622+1G>A | / | int17 | SPL | de novo | 1 | / | Pathogenic (PVS1-PM2-PP3) |
| 486 | sSoS | c.6258+1G>C | / | int21 | SPL | de novo | 1 | / | Pathogenic (PVS1-PM2-PP3) |
| 632 | sSoS | c.6259-2A>G | / | int21 | SPL | de novo | 1 | / | Pathogenic (PVS1-PM2-PP3) |
| 1312 † | sSoS | c.6463+5G>A | p=(?) | int 22 | SPL | de novo | 1 | / | Vus(PM2-PP3) |
| 1190 † | sSoS | c.927+5G>A | / | int3 | SPL | n.p. | 1 | / | Vus(PM2-PP2-PP3) |
| 679, 981 | sSoS/ sSoS | c.1236G>A | p.Lys412= | ex4 | SYN | np/np | 2 | PWWP1 | Vus (PM2-PP3) |
| 20-MOG-0010 | Overgrowth | c.3801G>C | p.(Val1267=) | ex6 | SYN | n.p. | 1 | / | Likely benign (PM2-BP4-BP7) |

| | | | | | | | | | |
|---|---|---|---|---|---|---|---|---|---|
| 1155 † | sSoS | c.5616_5618del | p.His1872_Ile1873delinsGln | ex17 | InDel | de novo | 1 | / | Vus (PM2-PM4-PP3) |
| 1318 † | sSoS | c.2504_2515del | p.(Gly835_Leu839delinsVal) | ex5 | InDel | de novo | 1 | / | Vus(PM2-PM4-PP3) |
| 468 | Overgrowth | c.4967-37_4967-34del | / | int13 | INT | paternal | 1 | / | Likely Benign (PM2-BP4) |
| 1141 | sSoS | c.5304-20C>T | / | int15 | INT | maternal | 1 | / | Likely Benign (PM2-BP4) |
| 1145 | sSoS | c.4642-37A>G | / | Int12 | INT | paternal | 1 | / | Likely Benign (PM2-BP4) |
| 1173 | sSoS | c.5623-137_5623-128del | p=(?) | int17 | INT | n.p. | 1 | / | Likely Benign (PM2-BP4) |

Variants identified in patients with a suspected sSoS or Overgrowth were predicted as pathogenic or likely pathogenic (n=95); as variants of unknown significance (VUS) (n=8), as likely benign (n=12). n.p.= not performed. MS: missense; MS-Cys: missense introducing/eliminating a Cysteine residue; NS: nonsense; FS: frameshift; SPL: splice-site; InDel: insertion/deletion; SYN: synonymous; INT: intronic; *: familial case; †: re-classified VUS; ° patients 1363 and 518 are compound heterozygous; /: patients with not specific clinical indication. In bolding we report the patients carrying two *NSD1* variants.

**Table S2. Sotos Collaborative group.**

| Institution | Name | email |
|---|---|---|
| Casa Sollievo della Sofferenza S.G Rotondo | Dr.M. Castori | m.castori@operapadrepio.it |
| U.O. Neuropsichiatria infantile | dr.ssa A.Pini | antonella.pini@ausl.bo.it |
| Ospedale S.M. delle Grazie | Dr. ssa Di Lucca | dilucca@virgilio.it |
| Ospedale policlinico di Catania | Dr.ssa T. Mattina | mattina@unict.it |
| AOU Meyer di Firenze | Dr.ssa E. Lapi | e.lapi@meyer.it |
| Ospedale riuniti di Bergamo | Dr.ssa M. Iascone | miascone@ospedaliriuniti.bergamo.it |
| Neuropsichiatria infantile Sassari | Dr.ssa G.Serra | giserra@uniss.it |
| Clinica Neurologica Bologna | Dr.ssa Cappanera | cappas@libero.it |
| Ospedala Bambino Gesu' | Dr.ssa MC. Digilio | mcristina.digilio@opbg.net |
| Ospedale di Varese | Dr. R. Casalone | rosario.casalone@ospedale.varese.it |
| IRCCS Stella Maris-Calambrone | Dr.A. Battaglia | agatino.battaglia@inpe.unipi.i |
| O.I Regina Margherita di Torino | Dr.ssa S. Vanelli | silvia.vanelli@unito.it |
| Gen Med Azienda ospedaliera pisana | Dr. E.Tarantino | e.tarantino@med.unipi.it |
| ASL BR1 UOS GENETICA | Dr. Lione | dr.lione@libero.it |
| UO Gen med policlinico S. Orsola Malpighi | Prof. M.Seri | marco.seri@unibo.it |
| Dip gen med Arcispedale S. Maria Nuova | Dr.ssa L. Garavelli | garavelli.livia@asmn.re.it |
| IRCCS "E MEDEA" la nostra famiglia | Dr.ssa R. Grasso | rita.grasso@bp.lnf.it |
| uo gen med AO BMM-RC reggio calabria | Dr. C. Mammi | corradomammi@tiscali.it |
| Ist Cli pediatrica di Cagliari | Dr.ssa L.Boccone | lboccone@mcweb.unica.it |
| Ambulatorio gen med ULSS 9 Treviso | Dr.ssa E.Frate | efrate@ulss.tv.it |
| U.O.gen med ULSS9 | Dr.ssa I.Turolla | lturolla@ulss.tv.it |
| Dip pediatria AO di Padova | Dr. M. Scarpa | maurizio.scarpa@unipd.it. |
| Ped Federico II | Dr. F. Majo | doctorfabius@yahoo.it |
| IST BESTA | Dr. S. D'Arrigo | darrigo@istituto-besta.it |
| Osp. GB. Rossi Verona | Prof. L. Tato' | luciano.tato@univr.it |
| Osp.Cardarelli,Napoli | Dr.ssa ML. Cavaliere | mlcavaliere@libero.it |
| Osp Le Scotte Siena | Dr.ssa C. Castagnini | cinzia_castagnini@yahoo.it |
| Gen clin Padova | Dr.ssa C. Daolio | cecilia.daolio@yahoo.it |
| UOC Gen Med Cardarelli | Dr.ssa P. Castelluccio | pia.cast@libero.it |
| Osp Gemelli | Dr G. Zampino | gzampino@rm.unicatt.it |
| pol. Monteluce Perugia | Prof. E.Donti | emilio.donti@gmail.com. |
| ped Ospedale Pavullo(MO) | Dr A. Forte | a.forte@ausl.mo.it |
| Pol Umberto I | Prof.L. Tarani | luigi.tarani@uniroma1.it |
| Osp Bambino Gesu' | Dr. S. Buonuomo | psabrina.buonuomo@opbg.net |
| ser.cons. gen.Azienda Osp Alto Adige | Dr. F.Stanzial | franco.stanzial@sabes.it |
| Neu Ped e malattie musc | Dr. M. Pedemonte | malattieneuromuscolari@gaslini.org |
| Neur. Ped. Federico II | Dr.E. Del Giudice | endelgiu@unina.it |
| UO gen med AOU Ferrara | Dr.ssa S. Bigoni | s.bigoni@ospfe.it |
| Osp microcitemico di Cagliari | Dr.ssa L. Boccone | lboccone@mcweb.unica.it |
| UOSD Malattie Rare - IRCCS Gaslini - Genova | Dr.ssa Di Rocco | majadirocco@gaslini.org |
| Osp Cardarelli Na | Dr. De Brasi | dantebrasi@hotmail.com |

| | | |
|---|---|---|
| U.O.C. Pediatria e Nido - P.O. di Scorrano | Dr. F. Nicastro | nicastrofrancesco@yahoo.it |
| Osp San Raffaele MI | Dr.ssa G. Garbetta | garbetta.gisella@hsr.it |
| IRCCS Stella Maris-Calambrone | Dr.ssa F. Peluso | francesca.peluso07@gmail.com |
